# Physicochemical Properties of UV-Irradiated, Biaxially Oriented PLA Tubular Scaffolds

**DOI:** 10.3390/polym15051097

**Published:** 2023-02-22

**Authors:** Pooja Bhati, Alok Srivastava, Ramya Ahuja, Pankaj Chauhan, Priya Vashisth, Naresh Bhatnagar

**Affiliations:** 1 Department of Mechanical Engineering, Indian Institute of Technology, Delhi 110016, India; 2 Department of Mechanical and Automation, Indira Gandhi Delhi Technical University for Women, Delhi 110006, India; 3 Homi Bhabha Cancer Hospital and Research Centre, Visakhapatnam 530053, India

**Keywords:** UV exposure, polymeric tubes, polymeric implants, bioactivity, polylactic acid, UV irradiation

## Abstract

PLA and its blends are the most extensively used materials for various biomedical applications such as scaffolds, implants, and other medical devices. The most extensively used method for tubular scaffold fabrication is by using the extrusion process. However, PLA scaffolds show limitations such as low mechanical strength as compared to metallic scaffolds and inferior bioactivities, limiting their clinical application. Thus, in order to improve the mechanical properties of tubular scaffolds, they were biaxially expanded, wherein the bioactivity can be improved by surface modifications using UV treatment. However, detailed studies are needed to study the effect of UV irradiation on the surface properties of biaxially expanded scaffolds. In this work, tubular scaffolds were fabricated using a novel single-step biaxial expansion process, and the surface properties of the tubular scaffolds after different durations of UV irradiation were evaluated. The results show that changes in the surface wettability of scaffolds were observed after 2 min of UV exposure, and wettability increased with the increased duration of UV exposure. FTIR and XPS results were in conjunction and showed the formation of oxygen-rich functional groups with the increased UV irradiation of the surface. AFM showed increased surface roughness with the increase in UV duration. However, it was observed that scaffold crystallinity first increased and then decreased with the UV exposure. This study provides a new and detailed insight into the surface modification of the PLA scaffolds using UV exposure.

## 1. Introduction

Polylactic acid has emerged as a green and clean biodegradable polymer produced from natural resources such as corn starch and sugar beet [1]. Due to the advantages associated with PLA such as bioresorbability, processability, biodegradation, and tailoring of the mechanical properties, it has received much-needed attention from researchers worldwide. The tailorable properties of PLA make it an in-demand polymer [2]. The PLA and its blends as biomedical scaffolds such as a stent and sutures have gained much-needed momentum at present [3]. This is due to its better biocompatibility, non-toxicity, and longer degradation time. Higher glass transition temperature (T_g_) makes PLA a sought-after biopolymer for medical and industrial use. However, the low surface bioactivity of PLA limits its application as a scaffold due to low cell adhesion and proliferation. Low cellular response on the PLA surface could cause slower endothelization of the scaffold or inflammation, and in extreme cases, even the rejection of the scaffold. Thus, for enhancing biocompatibility and the surface property of PLA, it can be improved using plasma treatment [3,4], ion and electron implantation [5], chemical modification [6], surface coating, surface roughening, and UV treatment. Among them, the UV treatment method is one of the easiest and most cost-effective methods to improve bioactivity in PLA scaffolds. The duration of UV treatment is based on the scaffold bulk properties and interaction between tissue cells and the polymer surface. Surface modification through UV exposure improves the cellular interaction of polymeric scaffolds [7]. However, UV treatment duration needs to be optimized. Thus, a polymeric scaffold that has superior mechanical properties and improved biocompatibility is the need of the hour.

Researchers have reported the effect of the long duration of UV treatment on the PLA [8,9,10]. However, long-duration exposure causes a drastic reduction in the mechanical properties of the PLA scaffolds due to bulk degradation of the polymer [11,12,13]. It also depends on the molecular weight, crystallinity, thickness, and surface properties. Thus, prolonged duration UV treatment can be avoided for surface modification of biaxially oriented PLA tubes. Therefore, in this study, the effect of short-duration UV treatment on the surface properties of PLA scaffolds was not investigated in detail and hence is needed to be adequately explored.

The objective of this work was to investigate the effect of UV irradiation treatment on the physicochemical properties and wettability of biaxially expanded PLA (PLA-UV) tubular scaffolds. For this purpose, the tube was fabricated by a novel biaxial extrusion setup developed in-house at IIT Delhi [14,15]. The fabricated tubular scaffolds were then irradiated by UV for different time intervals in order to understand the photochemical changes on the polymeric surface. 

## 2. Materials and Methods

PLA, Ingeo 4032D grade, density 1.24 g with 6.4 ± 0.3 MFI g/10 min at 210 °C/2.16 kg, M_w_ = 207 kDa [16] from Naturework LLC^TM^ (Plymouth, MN, USA), was the material.

### 2.1. PLA Tube Fabrication and Sample Preparation

PLA is hygroscopic; therefore, before extrusion of the tubular scaffolds, it was dried to have less than 250 ppm moisture to prevent hydrolysis. Thus, PLA was dried in the vacuum oven (−700 mm Hg) at a temperature of 50 °C for 12 to 16 hours and then at 70 °C for 3 h.

### 2.2. Fabrication of Biaxially Expanded Tubular Scaffolds

**Figure 1:**
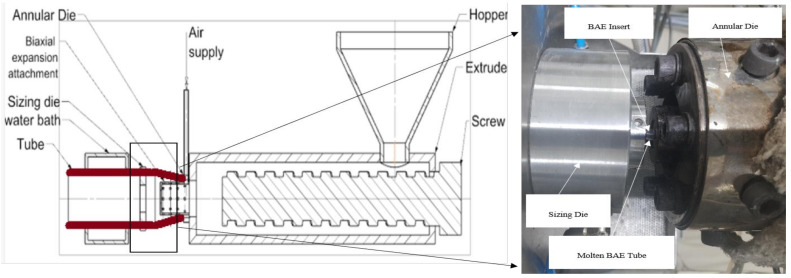
Biaxial expansion setup for tubular scaffold fabrication.

**Figure 2:**
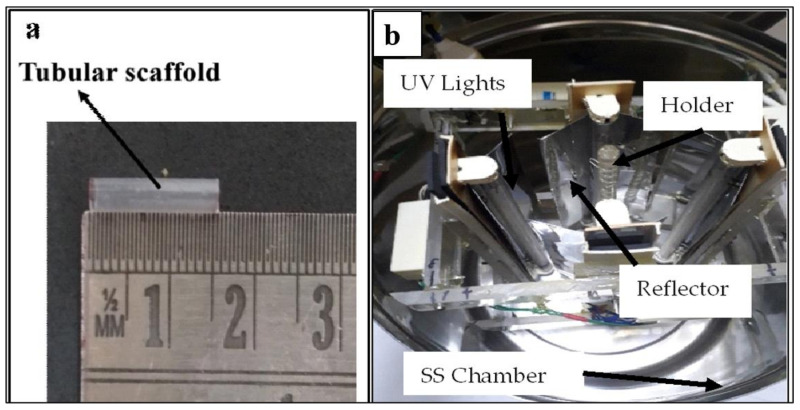
(**a**) The image showing fabricated tubular scaffold length and (**b**) an in-house manufactured UV treatment setup.

In brief, with the novel biaxial expansion of the tube, as shown in Figure 1, the continuous extrusion process was employed for the fabrication of tubular scaffolds. In this process, the extruded tube is drawn in radial as well as axial directions simultaneously as it comes out of the annular die. The forces are governed by caterpillar rpm, and compressed air is radially blown out from the die, as shown in Figure 1. This process was already established by Bhati et al. [14,15,17]. The tubes of 3 ± 0.05 mm diameter and a thickness of 120 ± 10 µm were extruded and cut into a sample specimen of 15 mm length, as shown in Figure 2a.

### 2.3. UV Irradiation

Figure 2a,b shows the fabricated tubular scaffold by the biaxial expansion method and an in-house manufactured UV treatment setup respectively.

In the circular UV light arrangement, the tubular sample was mounted at a distance (r) of 4 cm to achieve maximum exposure with minimal heat. All four 8W Philips© TUV 8W-G5FAM UV-C lamps (Philips Lighting N.V., Eindhoven, Netherlands) emitted light of the wavelength of 254 nm. Considering light as a point source, each bulb irradiance was one-fourth of the tube surface. According to Equation (1), [18,19] the average surface irradiance on a tube of 1 cm length was estimated to be 28 mW/cm^2^.

E = P/A = P/(4 × ᴨ × r^2^) (1)

E = irradiance of a surface (W/cm^2^), P is the radiant power (W); A is the surface area of a sphere of radius r (cm^2^).

The circular chamber was constructed from 3 mm-thick SS304 stainless steel. One window was cut into the chamber, and it was protected by a 20 mm-thick acrylic sheet. The biaxially expanded PLA tubes were exposed for 1 min, 2 min, 5 min, 10 min, and 15 min. All treatments were carried out in the air at room temperature. Additionally, as highlighted in Table 1, UV doses were calculated for each tube exposed for a particular time by using Equation (2) [20].

UV dose (J/cm^2^) = Irradiance (W/cm^2^) × Time (s) (2)

### 2.4. Characterization Techniques

**FTIR**: The chemical structure of the biaxially expanded and UV-irradiated PLA (PLA-UV) tubular scaffolds was investigated by using a Nicolet iS50 FTIR spectrometer (Thermo Fisher Scientific, Waltham, MA, USA) in ATR mode. Each FTIR spectrum was collected in a range of wavenumber 400–4000 cm^−1^ with a resolution of 4 cm^−1^ with a total of 32 scans.

**XPS**: X-ray photoelectron spectroscopy was utilized for the quantitative surface analysis of chemical changes introduced due to the UV irradiation on the PLA surface for different time durations. X-ray photoelectron spectroscopy (XPS) was used to measure the C1s and O1s of the untreated and UV treated PLA samples. The XPS system was equipped with an MgKα X-ray source.

**DSC**: Samples of 10–15 mg weight were used for the thermal analysis of the extruded PLA biaxially expanded scaffolds, which was performed on a DSC 6000 Perkin Elmer (Waltham, MA, USA). Indium was used for the reference for the temperature calibration. For DSC, the sample was heated from 25 to 180 °C, and the rate of heating was maintained at 10 °C/min.

**TGA**: DSC 6000 Perkin Elmer (Waltham, MA, USA) was employed to assess the thermal decomposition of the scaffolds. Samples were heated from 10° to 400 °C. The heating rate was maintained at 10 °C/min. The initial degradation temperature at 10% material weight loss (T10), weight loss, and the end of the degradation were observed for the analysis.

**Mechanical testing**: Radial compressive testing was performed on the scaffolds to access the effect of UV irradiation on the mechanical performance. The test was performed on the Instron 5582 machine using Bluehill software version V4 as per ISO 25539-2, which is described in detail elsewhere [15]. The crosshead speed was kept at 1 mm/min.

**WCA**: The apparent static water contact angle (WCA) for measuring surface wettability was determined by using the sessile drop method. Deionized water droplets of 0.5 μL volume were placed on the tube. The WCA on the samples was evaluated by analyzing the images using ImageJ software Version 1.51.

**SEM**: SEM (Zeiss EVO 50, Oberkochen, Germany) was used to study the effect of UV exposure on surface morphology. SEM micrographs for a small portion of gold-coated tubular scaffolds were obtained and studied for the detailed study.

**AFM**: Surface morphology was studied by AFM using a digital instrument, NanoScope IIIa (Bruker, Billerica, MA, USA), in tapping mode. The root mean square (RMS value) was calculated before and after UV treatment.

All experiments were performed for sample size *n* = 5. Statistical analysis was performed using MS Excel for Student’s *t*-tests, and *p*-values < 0.05 were considered statistically significant.

## 3. Results and Discussion

### 3.1. Physicochemical Properties of PLA-UV

**Figure 3:**
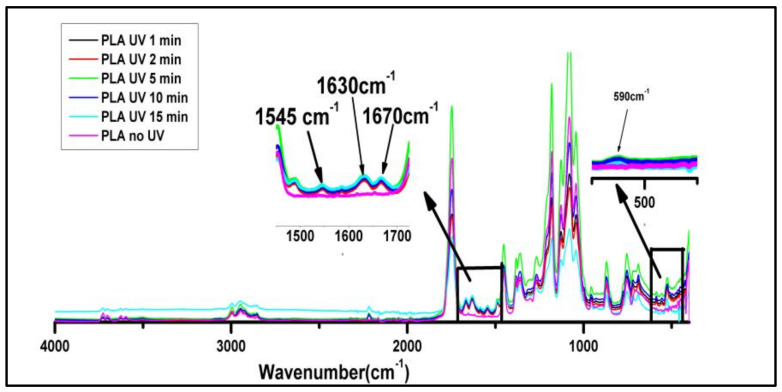
FTIR spectra of PLA tubular scaffolds before and after UV irradiation.

The FTIR spectra were collected from the PLA-UV tubular scaffolds irradiated for a different time duration, as shown in Figure 3. The characteristic bands of pure PLA present in the spectra are given in Table 1. A broad absorption band in the hydroxyl region with maxima at 3600 cm^−1^ was observed, confirming products such as peroxides or alcohols. It was noted that after UV irradiation, a new peak in the region from 1520 to 1680 cm^−1^ was observed whose maxima were at 1665 and 1630 cm^−1,^ respectively (two maxima). These peaks could have been due to the new carbonyl groups formed by the *α* cleavage of the ester bond in the PLA backbone with the partial abstraction of the hydrogen. Less intensive peaks were also observed between the 1600 and 1650 cm^−1^ wavenumber, which could have been due to per-ester or per-acid and the presence of the double bond functional groups. As reported by Bocchini et al. and Gardette et al. [21,22], the formation of an anhydride group was at 1845 cm^−1^, but it was absent and could have been due to the short time UV treatment, or another possibility could be ozone formation under the UV light that initiates faster oxidation of PLA [8,23]. It was revealed that UV light breaks down the oxygen molecules present inside the chamber into free radicals, which leads to the de-esterification of PLA. This result shows that with the increase in the UV irradiation duration, the intensity of the various spectral bands also increased, but after 5 min of UV treatment, it was reduced. This implies that the chain scission of the PLA-UV polymer started after 5 min of UV exposure. The drop of the band intensities at 10 min and 15 min UV exposure indicated the polymer photolysis process.

Moreover, it can be confirmed as a new weak peak at 525 cm^−1^ was observed due to C-C bonding [24]. Another limitation beyond 10 min of UV exposure was that the OH stretching at 3000 cm^−1^ became much broader and its maximum also shifted towards the lower wavelength. This phenomenon is attributed to the change in hydroxyl groups or due to UV degradation and photo-oxidation [25]. Table 2 Shows the wavenumbers and functional groups of the functional groups present in PLA.

### 3.2. XPS

**Figure 4:**
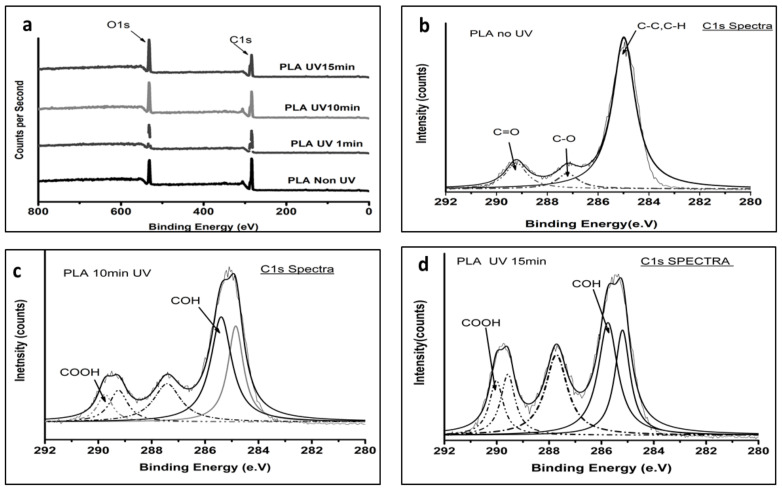
(**a**) Low-resolution XPS scan of UV-irradiated PLA for different time durations. (**b**–**d**) C1s XPS detailed spectrum of non-UV irradiated PLA compared to 10- and 15-min UV irradiated PLA, respectively.

The low-resolution XPS spectra were obtained before and after UV irradiation on the PLA-UV tubes, which demonstrated the detailed chemical structure of the polymeric surface. As expected (Figure 4a), only carbon and oxygen peaks were present in the PLA-UV. According to the literature [9,27,28], the PLA chemical structure contains two prominent peaks at binding energy, 284.8 eV and 533 eV, which correspond to C(1s) and O(1s), respectively. It was observed that with an increase in the UV duration, the intensity of the O(1s) peak increased and the C(1s) peak reduced. If the peak increased, it corresponded to the number of atoms of that particular oxidation state, also increased. Thus, the high peak intensity of oxygen implies the oxidation of the PLA surface.

The high-resolution C1s spectra of UV-irradiated samples 10 and 15 min were compared with the untreated sample as observed in Figure 4b–d. The C1s peaks were deconvoluted to obtain a deep understanding of the surficial carbon atom. The peak at a binding energy of 284.8 eV corresponded to the sp^2^ and sp^3^ states of carbon atoms, which were bonded either to carbon or hydrogen (C-C, C-H). The UV irradiation time was directly proportional to the shift in the peak 284.6 eV, which can be related to the high binding energy. A minute shift for 10 min UV and 15 min UV was observed towards the higher binding energy. This was due to changes in the chemical nature of the surrounding atoms.

**Figure 5:**
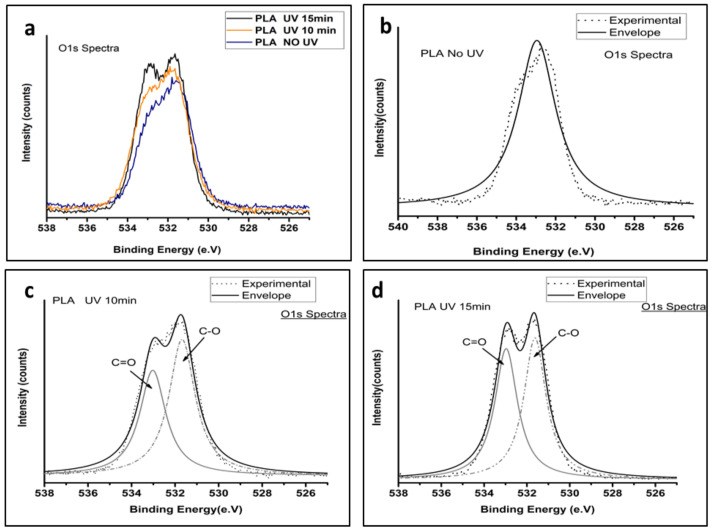
O1s spectra of PLA-UV: (**a**) Low-resolution spectra of irradiated BE-PLA. (**b**–**d**) High-resolution O1s spectra of non-irradiated PLA compared with UV 10 and 15 min treated samples.

Furthermore, Figure 5a shows the O1s spectra of the non-irradiated and 10- and 15-min UV irradiated PLA tubular scaffold. It validated that the O1s peak intensity increased with an increase in UV irradiation time. Moreover, peak shouldering was observed in the samples irradiated for longer durations. A detailed study of the spectra revealed that in non-irradiated PLA scaffolds, only a single peak was seen at a binding energy of 532.9 ± 0.01 eV, which was attributed to oxygen atoms of the ether links [29].

As the exposure time reached 10 and 15 min, a new peak at 533.02 ± 0.02 eV was observed with peak shouldering (Figure 5c,d) related to oxygen atoms of carbonyl groups, as shown in Figure 5b. It led to higher functional oxygen groups on the scaffold surface, which could be the main reason for enhanced surface hydrophilicity.

Furthermore, an increase in UV irradiation duration resulted in new group formation on the scaffold surface belonging to COOH and COH groups. The formation of such functional groups was found to be responsible for the high wettability of the scaffolds.

These results agree with the results obtained from the FTIR spectra and provide a reasonable and firm explanation for the decreases in the static water contact angle of the PLA-UV scaffold surface with increased UV duration. Similarly, the peak intensity of the CH_3_ group decreased with irradiation time. It indicates that the photodegradation mechanism caused breakage of the C-C bond in the PLA polymer chain, which then can react with oxygen and give rise to polar functionality.

### 3.3. Thermal Stability

**Figure 6:**
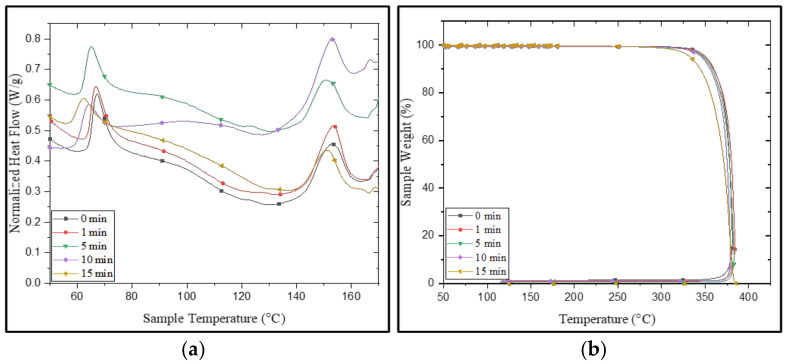
(**a**) DSC and (**b**) TGA of PLA non-UV- and UV-irradiated tubes.

The DSC thermograph (Figure 6a) after UV treatment negatively affected the glass transition temperature (T_g_) and melting temperature (T_m_) due to the depolymerization of the polymeric chain. However, in between 1 to 5 min of UV action, the crystallinity was enhanced, as a few minutes of exposure broke down the amorphous phase on the surface, resulting in an increase in the number of well-ordered PLA molecules. Hence, the degree (Xc) of crystallinity increased. However, crystallinity again decreased as the treatment increased; this may have been due to the deep penetration of UV that dominated the scissoring of the polymeric chain.

The bi-axial stretching with a smaller dosage of UV exposure increased the crystallinity. Thermographs (Figure 6b) also demonstrated that UV exposure can induce small nucleating sites, which were confirmed by the TGA graph; the melting temperature decreased after irradiation [30]. After 5 min of UV radiation, the temperature after 10% of degradation (T_10_) decreased from 360 to 357 °C; after that, it was significantly reduced to 342 °C after 15 min. The decrease in the degradation temperature can be explained by the shortening of macro chains due to oxidative degradation [12]. After 15 min of exposure to UV radiation, the PLA became brittle and started cracking due to photo-oxidation. The result indicated that PLA showed a minimum shield toward UV irradiation; however, a small duration of UV treatment (<5 min) on the PLA surface showed positive effects on physicochemical properties. Table 3 shows the different properties of UV irradiated PLA tubular scaffolds.

### 3.4. Mechanical Characterization—Radial Compressive Testing

**Figure 7:**
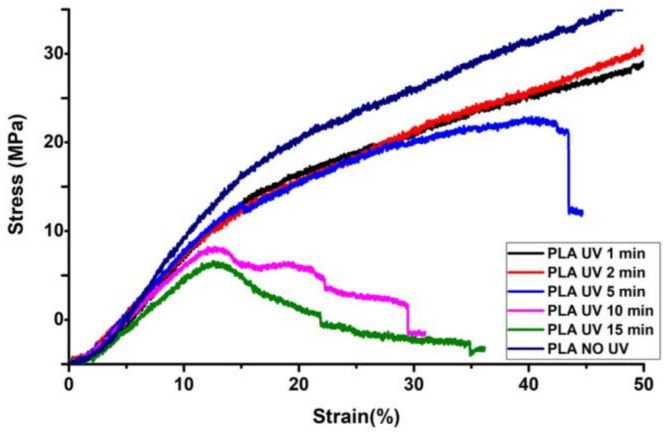
Radial compressive stress–strain curve before and after UV exposure.

Figure 7 shows the compressive stress-strain (%) curve of the tubular scaffold before and after UV exposure. From the curve, it can be inferred that nonirradiated PLA and PLA scaffolds UV irradiated until 5 min showed brittle behavior. However, as the UV irradiation increased to 10 and 15 min, the PLA behavior changed from brittle to ductile. Moreover, the compressive strength of the PLA tubular scaffolds decreased gradually as the UV exposure duration changed for 1, 2, and 5 min. Significant changes in compressive strength were observed for the specimens that were UV-irradiated for 10 and 15 min. The tensile strength of the 1- and 2-minute UV-irradiated samples did not have a significant variation as compared to nonirradiated samples. The radial compressive strength of 1-, 2-, and 5-minute irradiated samples reduced by 7.03, 9.01, and 11.32%, respectively. However, a drastic reduction in radial compressive strength was noticed for the samples irradiated for 10 and 15 min, and the compressive strength of the samples decreased by 31.7 and 38.7%, respectively.

Nevertheless, the compressive strength showed a decreasing trend with an increase in UV exposure duration, which could have been due to induced changes to the greater depth into the scaffold surface.

### 3.5. Surface Wettability

**Figure 8:**
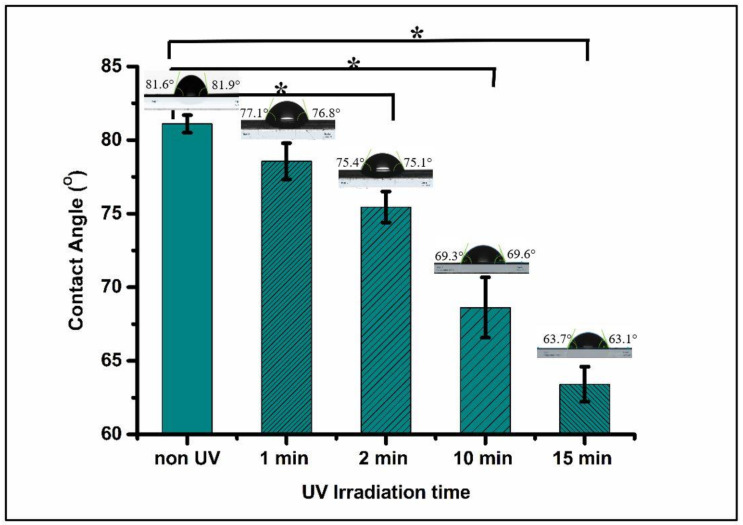
Water contact angle of PLA before and after UV irradiation (* means significantly different, *p* < 0.05).

The wettability studies were carried out to explore the effect on surface hydrophilicity after UV irradiation. The wettability of a scaffold surface has a strong positive influence on cell adhesion and growth [7]. It has been observed that with increasing UV irradiation, the surface hydrophilicity of all the tubular scaffolds increases. Initially, the untreated PLA scaffold WCA was 81°, which indicates the surface was hydrophobic. However, after 1 min of UV treatment, the WCA decreased to 78.5°, and further exposure of PLA tubular scaffolds to UV irradiation reduced the WCA, indicating better wettability. Similar results were obtained by Bhati et al., wherein the UV-irradiated tubular scaffolds exhibited better biocompatibility than non-irradiated tubes (Figure 8) [29].

### 3.6. Morphological Characterization

#### 3.6.1. SEM Analysis

**Figure 9:**
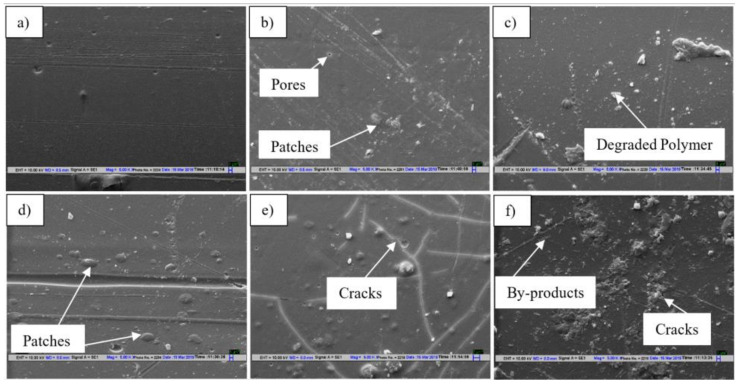
SEM micrographs of PLA UV-treated scaffolds at different time intervals: (**a**) 0 min, (**b**) 1 min, (**c**) 2 min, (**d**) 5 min, (**e**) 10 min, and (**f**) 15 min.

SEM images of PLA tubes before and after UV treatment are shown in Figure 9a. It was observed that after 1 min (Figure 9b) of exposure, a few small patches and pores were observed on the surface, which became more homogenously distributed as the treatment duration increased. Next, from 2 to 5 min (Figure 9c,d), patches with some products were seen on the tube surface, which can be correlated with photodegradation. However, significant changes were observed from 5 to 10 min, as more patches and small cracks were seen on the PLA surfaces, as shown by SEM images in Figure 9e. As a result, this increased surface roughness compared to untreated PLA. However, after 15 min (Figure 9f), the amount of by-products was substantially higher, and cracks became deeper due to the de-esterification of PLA. Finally, due to severe deep microcracks and decomposition, tubes lost their structural integrity, resulting in brittle failure [29,30].

#### 3.6.2. AFM Study

**Figure 10:**
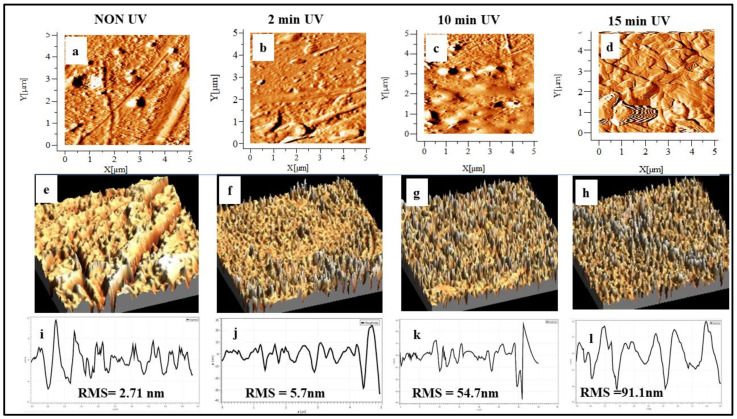
AFM images of PLA tubular scaffolds before and after UV irradiation.

AFM is used for assessing the surface roughness and surface topography of the tubular surfaces before and after UV treatment. Figure 10 shows the AFM images of PLA tubular scaffolds after UV exposure for 0, 1, 2, 5, 10, and 15 min. The results illustrate that the untreated PLA had a smooth surface (RMS = 2.71 nm)

As the UV exposure increased to 2 min, there were no significant changes in the surface morphology and roughness. However, the surface topography was considerably high as UV duration increased after 10 min. The results revealed UV exposure directly in proportion to the surface roughness. The increased RMS could have been due to the formation and accumulation of the photodegraded products on the surface, as can be observed in Figure 10c,d [9]. The 10 min UV-treated PLA scaffold AFM image showed a higher number of granular structures on the surface, resulting in undulation on the surface [30]. Such surface morphology could be due to the photodecomposition of long-chain macromolecules. The undulations are primarily oxidized of low-molecular-weight photo-degraded products that are formed due to the longer duration of UV exposure.

This can be correlated with the FTIR study where the large formation of new carbonyl groups was observed on the surface after long UV exposure. These undulations could be due to oxygen-containing functional groups formed due to prolonged UV exposure.

Similarly, the decrease in the water contact angle with an increase in UV exposure time also proved that oxygen-containing functional groups are significantly increased on the surface after 5 min of UV exposure. As the UV duration changed from 10 to 15 min, the roughness was continuously increased, leading to higher surface wettability with micro/nano pits. Similar findings from other researchers reported that surface hydrophilicity increased with UV treatment [31,32,33,34,35].

The surface roughness plays an important role, as the first interaction of the cell occurs with the scaffold. The cellular response to the implanted scaffolds is governed by the hydrophilicity, morphological properties, and chemical properties of the surface. Thus, surface modification is a crucial process for making the scaffold surfaces more conducive for the cellular response. To improve and offer a surface of greater bio-affinity and biocompatibility for cellular interaction, we modified it using ultraviolet (UV) irradiation, since this technique, in comparison with others, such as plasma and wet chemicals, has low cost, no use of solvents, and easy handling.

## 4. Conclusions

The physicochemical properties of UV-treated PLA scaffolds were changed due to photodegradation. During the UV treatment, the formation of free radical oxygen inside the closed chamber initiated faster degradation that led to the development of the higher functional oxygen groups. These degraded by-products such as hydroxyls, carbonyls, carboxylic acids, and peroxides were accumulated on the surface of the scaffolds.

Similarly, the thermal analysis revealed that UV exposure beyond 5 min increased the degradation rate, which explains the shortening of macro chains due to photo-oxidation. Hence, the interaction of oxygenated by-products present on the surface encouraged the wettability of the scaffolds. Additionally, the surface roughness also increased with UV irradiation—a few small patches were noted at 5 min, which ultimately led to brille failure at 15 min.

Finally, a short duration (<5 min) of UV treatment on the PLA scaffolds was shown to positively modify the surface properties, which can be beneficial for tissue engineering applications.

## Figures and Tables

**Table 1:** Amount of UV doses on the tubes w.r.t. UV irradiance time.

**Time (min)**	1	2	5	10	15
**UV doses (J/cm^2^)**	1887	3774	9436	18,872	28,308

**Table 2:** Wavenumber and functional groups present in PLA [26].

Wavenumber (cm^−1^)	Functional Group
1750	(C=O) stretching
1450	(C-H) stretching
1180	C-C(O)-C stretching
1080	(C-O) stretching
867	(C-COO) stretching

**Table 3:** Different properties of UV-treated PLA samples.

UV-Treated PLA Sample (min)	T_g_ (°C)	T_m_ (°C)	ΔH_m_ (W/g)	χ_c_ (%)	T_10_ (°C)
0	67.1	152.7	15.1	16.9	360.01
1	66.8	152.3	18.1	20.3	359.93
5	65.1	151.9	16.0	17.9	357.7
10	64.1	151.5	14.9	16.7	355.85
15	62.61	150.2	14.1	15.8	342.66

## Data Availability

The data presented in this study are available on request from thecorresponding author.
